# Association between vaccination and the risk of central demyelination: results from a case-referent study

**DOI:** 10.1007/s00415-023-11822-y

**Published:** 2023-06-23

**Authors:** Lamiae Grimaldi-Bensouda, Caroline Papeix, Yann Hamon, Jacques Benichou, Lucien Abenhaim

**Affiliations:** 1 The PGRx Study Group, Paris, France; 2 Pharmacology Department, Hospital Group Paris-Saclay, Assistance Publique-Hôpitaux de Paris, Garches, France; 3 University of Versailles-Paris Saclay, Montigny Le Bretonneux, France; 4 Inserm U 1018 CESP, Villejuif, France; 5 Neurology Department of Hospital foundation A de Rothschild, Paris, France; 6 Paris-Cité University, Paris, France; 7 RESAL, LA-SER Group, Paris, France; 8 Department of Biostatistics and Clinical Research, CHU Rouen, 76000 Rouen, France; 9 Université de Rouen-Normandie, Rouen, France; 10 Department of Epidemiology, London School of Hygiene and Tropical Medicine, London, UK; 11 Centre for Risk Research Inc., Montreal, Canada

**Keywords:** Central demyelination, Vaccines, Multiple sclerosis, Case-referent study

## Abstract

**Background:**

Few studies documented the potential association between vaccination and the risk of central demyelination (CD). Specifically, anti-hepatitis B and anti-human papillomavirus (HPV) vaccines have been the subject of distrust with regard to their implication to trigger CD.

**Methods:**

From a systematic national registry, patients with first signs of CD (cases) were identified and documented for their exposure to vaccination up to 24 months before the first signs occurred. This exposure was compared to that of a representative sample of general practice patients without a history of CD, randomly selected from a national registry (referents). CD cases were 2:1 matched on age, sex, index date (ID), and region of residence. Vaccines against influenza, HPV, hepatitis B and diphtheria–tetanus–pertussis–poliomyelitis–haemophilus (DTPPHae) were considered. Associations between vaccination and CD were assessed using multivariate conditional logistic regressions, controlled for confounding factors.

**Findings:**

564 CD cases were matched to 1,128 randomly selected referents (age range: 2–79 years old). Overall, 123 (22%) CD cases and 320 (28%) referents had received at least one vaccine within 24 months before ID. Adjusted odds ratios (ORs) for any vaccination were 0.69, 95% confidence interval (CI) [0.54–0.88] with respect to any CD first signs, 0.68 [0.51–0.90] for myelitis and 0.70 [0.42–1.17] for optic neuritis. Adjusted ORs for any CD first signs were 1.02 [0.71–1.47] for influenza vaccine (administered in 9.6% of cases and 10.4% of referents) and 0.72 [0.53–0.99] for DTPPHae vaccine (administered in 10.8% of cases and 14.5% of referents). Vaccines against hepatitis B and HPV were only administered in 1.1% and 1.2% of cases and in 2.9% and 3.2% of referents respectively, which statistically explained the point estimates < 1 (ORs of 0.39 [0.16–0.94] and of 0.32 [0.13–0.80]).

**Interpretation:**

No increased risk of CD incidence was observed amongst vaccinated patients. Lower rates of vaccination against hepatitis B and HPV observed in patients with CD compared to referents may be due to the reluctance of physicians to vaccinate patients considered at risk of CD.

**Supplementary Information:**

The online version contains supplementary material available at 10.1007/s00415-023-11822-y.

## Introduction

Central demyelination (CD) is an immune-mediated disorder of the central nervous system, which is manifested by a variety of non-specific and specific neurological symptoms that can precede the diagnosis of the disease by a few weeks to several years. The most common disorder is multiple sclerosis (MS) [1] whose first signs may be manifested by optic neuritis in 20% of cases [2]. The precise aetiology of this disorder still remains largely unclear [3, 4], yet it is commonly agreed that the onset of the disease is influenced by genetic and environmental factors [5–7]. The importance of the environment has been highlighted by a study showing lack of genetic differences between monozygotic twins discordant for MS [8]. Evidence for involvement of the environment in MS development also comes from a variety of epidemiological studies linking MS to latitude [9, 10], vitamin D levels [11, 12], smoking status [5, 13–15] and exposure to certain infections (mainly Epstein–Barr virus) [5, 16, 17]. Some studies have suggested that immunization could play a role in the development of auto-immune disorders including CD, notably MS [18, 19]. It has been suggested that immune responses generated by infections or vaccination may cause trigger or exacerbate central nervous system autoimmunity in susceptible individuals [20, 21]. In some countries, particularly in France, pharmacovigilance reports have recorded a sizeable number of CD cases closely following vaccination, especially with the anti-hepatitis B vaccine (HBV) [22, 23]. Recent meta-analyses of observational studies explored the association between vaccines and the risk of CD [24, 25]. Most of these studies focussed on the risk of CD or MS following anti-hepatitis B vaccine or anti-human papilloma virus (HPV) vaccine, with controversial results. Indeed, most of these studies showed no association [26–30], whilst two studies suggested an increased risk of CD or MS after anti-hepatitis B or anti-HPV vaccines [31, 32], leading to vaccine distrust in some countries. However, some studies on the risk of CD associated with vaccines presenting major flaws or biases, for example, using the date of CD diagnosis or an imprecise date of first signs of CD. In the light of the persisting doubts about a possible link between vaccination and the risk of incident cases of CD, notably MS, a comprehensive case-referent epidemiological study was undertaken to evaluate the risk of CD following vaccination.

## Methods

Using two national registries, a case-referent study was conducted in France under the supervision of an independent Scientific Committee and following the guidelines for Good Pharmacoepidemiology Practices (GPP). The reporting followed the criteria of the STROBE Statement [33].

### Study population

Cases and referents were recruited prospectively using the PGRx information system. CD cases were identified within a systematic national registry (« PGRx-Demy» registry) assembled by 43 neurology centres specialized in central demyelination from across France, between December 2007 and July 2014. An on-site audit of recruitment and data quality was performed over a 2-month period. Referent-patients were recruited by a network of 406 general practitioners (GPs) participating in the “PGRx-GP” registry. Patients were offered participation regardless of their reason for consulting a physician. From these registries, patients were retained for this study if they met the following inclusion criteria: (1) aged ≥ 2 years; (2) living in France; (3) able to undergo a telephone interview in French (the participant himself/herself or his/her parents); and (4) consenting to participate to the study (parental consent for minor participants).

### Central demyelination cases

Patients with a first lifetime sign of demyelination affecting the brain, spinal cord or optic nerve were identified from « PGRx-Demy» registry and documented for their exposure to common vaccines up to 24 months before the first sign occurred. CD was defined according to international conventions [34]. Patients with a personal history of CD at inclusion or presenting other disease that could explain the current episode suggestive of CD were excluded.

### Referents and matching

A representative sample of GP patients without a history of MS or CD was randomly selected from the « PGRx-GP» registry (i.e. referents’ registry).

Two referents were matched to each CD case on age (the nearest age under 1 year of difference), sex, index date (first sign in cases and date of recruitment in referents; difference not exceeding 3 months) and region of residence (Northern or Southern France).

### Exposure to vaccination

In cases and referents, information on all medications (including vaccines and over-the-counter medication) used in the 24 months preceding the index date was collected through a structured and standardized telephone interview. Interviews lasted for 1 h and were conducted within 45 days of recruitment by trained interviewers using the previously validated Progressive Assisted Backward Active Recall (PABAR) method [35, 36]. Parents of children younger than 18 years old were interviewed. To facilitate remembering the medications taken, an interview guide was provided to participants or their parents prior to the telephone interview. Patients and referents were asked to report any medications and vaccines received, even when not listed. Exposure to vaccines was also systematically informed by physicians, in cases and referents and reported using an electronic case notification form. Exposure to vaccines was considered as “certain” when either patients or their physicians provided a tangible proof for vaccination: vaccine batch number, vaccination booklet, prescription noted in health medical record, pharmacist's report or any other type of vaccination certificate. Vaccines against influenza, HPV, diphtheria–tetanus–pertussis–poliomyelitis–haemophilus (DTPPHae) and hepatitis B were considered.

### Time windows

The main time windows for exposure to vaccines (any vaccines) prior to the index date were 2, 6 and 24 months. These time windows were determined by the independent Scientific Committees to best capture potential associations between vaccines and the occurrence of CD.

### Statistical analysis

For each variable, if < 5% of cases and < 5% of referents had a missing value, the missing values were imputed, using simple imputation with the median (for continuous variables) or the mode (for categorical variables) of non-missing observations, collected in each of the cases and referents strata respectively. If ≥ 5% of cases or ≥ 5% of referents had a missing value for a categorical variable, a ‘missing’ classification was reported as a separate category of the covariate. This was the case for the variables “family history of auto-immune diseases” and “region of residence”. This level of missingness was not observed for any quantitative variable.

In view of the matching of referents to CD cases on age, sex, index date and region of residence, conditional logistic regression was used to assess potential associations of vaccination (all vaccines and for each vaccine separately) with CD, for (1) all CD, (2) optic neuritis and (3) myelitis. The following potential confounders of the relation between the exposure to vaccines and the risk of CD, defined based on prior knowledge and clinical experience, were adjusted for in order to obtain adjusted matched odds ratios and corresponding 95% confidence intervals (CI): family history of auto-immune disorders, region of residence, smoking status and co-vaccinations.

For all statistical tests, significance was assessed with respect to the two-sided 0.05 level. Models were also stratified by age groups (< 25 years old, 25–44 years old, ≥ 45 years old).

Statistical analyses were performed using SAS software version 9.4 (SAS Institute Inc., Cary, NC, USA).

### Ethics committee approval

The study protocol was approved by the Ethics Review Committee of Paris-Ile de France III (Comité de Protection des Personnes Ile de France III) and data collection was approved by the French data protection authority (“*Commission Nationale de l’Informatique et des Libertés*”; CNIL). All participants or their parents (if aged < 18 years) gave their written informed consent. Physicians, but not patients, were compensated for inviting and recruiting patients.

## Results

### Study population

**Fig. 1:**
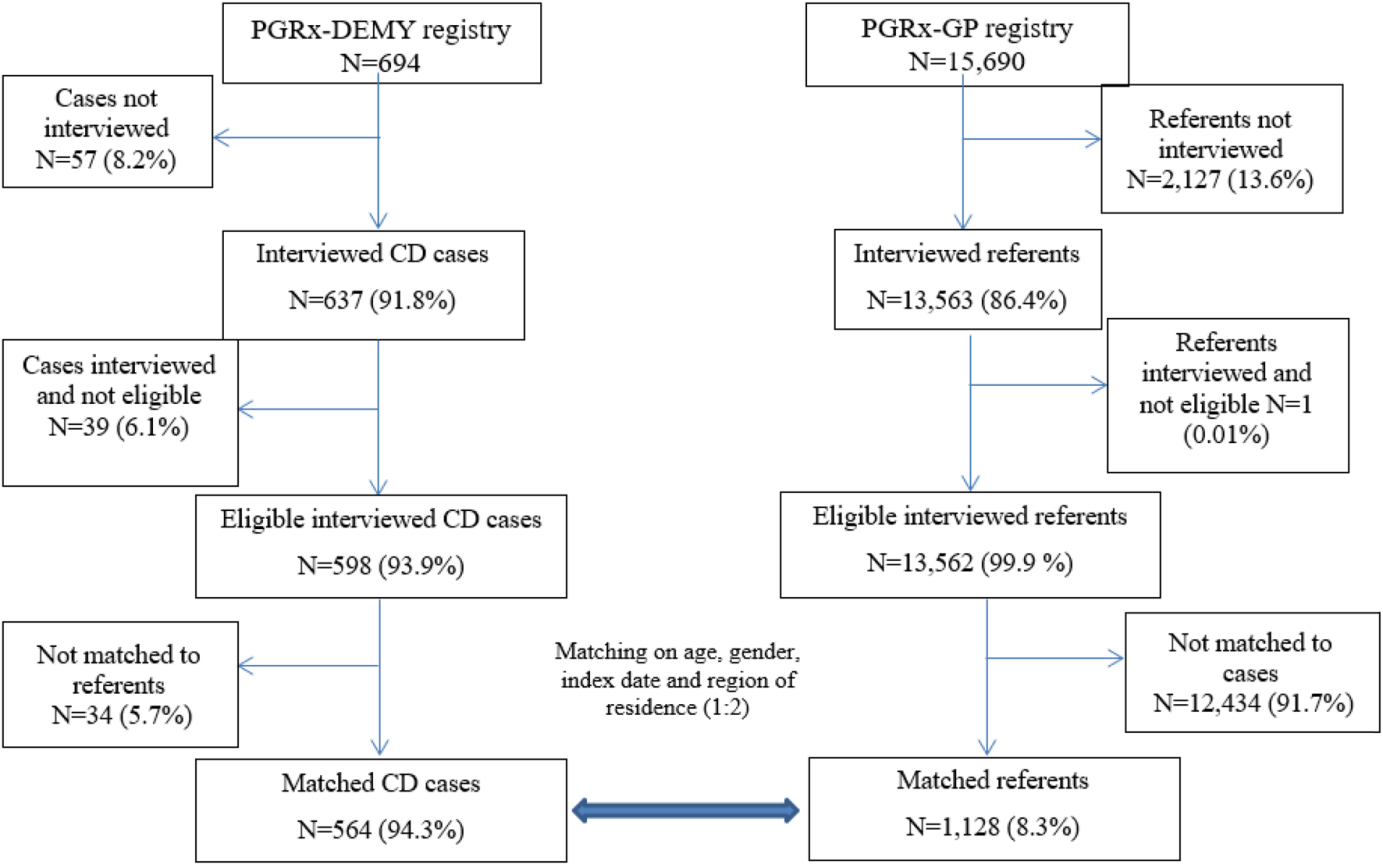
Flow chart of patients’ recruitment in the central demyelination and general practice registries

Amongst the 694 incident CD cases identified in the systematic PGRx-Demy registry and 15 690 referents in the PGRx-GP registry, 598 CD cases and 13,562 referents met inclusion criteria for this study. Moreover, 564 CD cases were matched to 1128 randomly selected referent patients without a history of demyelination on age, sex, index date (first sign in cases) and region of residence (Northern or Southern France) (Fig. 1). The CD cases seemed to be more likely to have a family history of auto-immune diseases, to be smokers and alcohol drinkers than the referents (Table 1).

**Table 1:** Characteristics and distribution of risk factors in cases and matched referents

	Cases	Matched referents
	*N = *564 *n* (%)	*N = *1128 *n* (%)
Familial history of AID (excluding T1D)		
Yes	3 (0.5%)	1 (0.1%)
No	561 (99.5%)	1127 (99.9%)
Region of residence		
Northern or Southern France	347 (61.5%)	740 (65.6%)
Others, mixed or missing	217 (38.5%)	388 (34.4%)
Smoking status		
Smoker	249 (44.7%)	462 (41.3%)
Former smoker (has stopped smoking for ≥ 1 year)	94 (16.9%)	164 (14.6%)
Never smoked	214 (38.4%)	493 (44.1%)
Missing/Chose not to answer	7	9
Alcohol consumption (in a normal week before ID)		
Daily or almost daily	33 (5.9%)	52 (4.7%)
A few times per week	74 (13.3%)	150 (13.4%)
Occasionally or never	450 (80.8%)	916 (81.9%)
Missing/chose not to answer	7	10
Co-vaccinations		
Certain	123 (21.8%)	320 (28.4%)
Uncertain	10 (1.8%)	43 (3.8%)
Not exposed	431 (76.4%)	765 (67.8%)

*AID* autoimmune disease; *ID* index date; *T1D* type 1 diabetes

### Vaccines and the risk of central demyelination

**Fig. 2:**
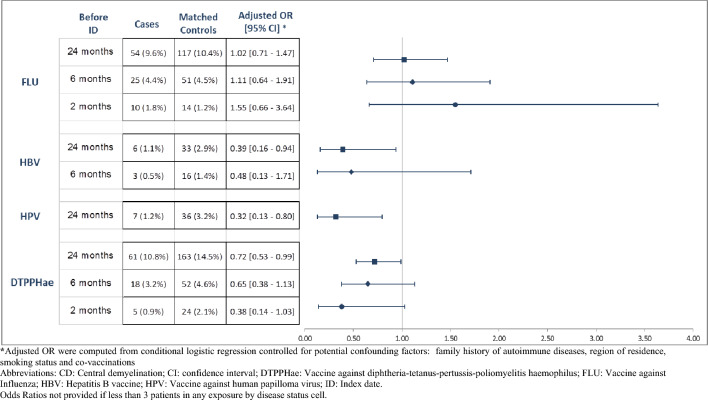
Association between vaccines and central demyelination, before its first sign according to type of vaccine

Overall, 123 (22%) cases and 320 (28%) referents had received at least one vaccine within the 24 months before the index date. Adjusted odds ratios (OR) were 0.69; 95%CI [0.54–0.88] for any CD first sign, 0.68; 95%CI [0.51–0.90] for myelitis and 0.70; 95%CI [0.42–1.17] for optic neuritis. The adjusted ORs for the other time windows (6 months and 2 months) were very similar to those of the 24-month time window (Table 2). According to vaccine type, adjusted ORs for any CD were 0.39; 95%CI [0.16–0.94] for anti-hepatitis B vaccine, 0.32; 95%CI [0.13–0.80] for anti-HPV vaccine, 1.02; 95%CI [0.71–1.47] for influenza vaccine and 0.72; 95%CI [0.53–0.99] for DTPPHae vaccine in time windows of 24 months. Similar results were observed for the 6-month and 2-month time windows when it was possible to estimate the adjusted ORs in a reliable manner (e.g. adjusted ORs were not estimated if there were fewer than 3 patients in any exposure by disease status cell) (Fig. 2). When 2-month and 6-month time windows were used, a positive non-significant association was found between the influenza vaccine and the risk of CD (OR = 1.55; 95% CI [0.66–3.64] and OR = 1.11; 95%CI [0.64 – 1.91], respectively). The stratification by age groups yielded similar trends (Supplementary Materials, Table S1).

**Table 2:** Association between any vaccine and central demyelination, optic neuritis or myelitis before their first signs

Any vaccine	Cases *n* (%)	Matched r ferents *n* (%)	Matched adjusted OR [95% CI]^a^
Central demyelination	*n = *564	*n = *1,128	
Vaccine within: 24 months before ID	123 (21.8%)	320 (28.4%)	0.69 [0.54–0.88]
6 months before ID	52 (9.2%)	136 (12.1%)	0.78 [0.55–1.11]
2 months before ID	19 (3.4%)	52 (4.6%)	0.74 [0.43–1.28]
Optic neuritis	*n = *132	*n = *264	
Vaccine within: 24 months before ID	27 (20.5%)	68 (25.8%)	0.70 [0.42–1.17]
6 months before ID	12 (9.1%)	26 (9.8%)	1.00 [0.48–2.07]
2 months before ID	4 (3.0%)	11 (4.2%)	0.82 [0.25–2.67]
Myelitis	*n = *432	*n = *864	
Vaccine within: 24 months before ID	96 (22.2%)	252 (29.2%)	0.68 [0.51–0.90]
6 months before ID	40 (9.3%)	110 (12.7%)	0.74 [0.50–1.10]
2 months before ID	15 (3.5%)	41 (4.7%)	0.74 [0.40–1.37]

*CI* confidence interval; *ID* index date

^a^Adjusted OR were computed from conditional logistic regression controlled for family history of autoimmune diseases, region of residence, smoking status and co-vaccinations

## Discussion

A national observational case-referent study was conducted to assess the association between vaccination and CD, considering at risk periods before CD first signs. Our results showed no increase in the risk of developing CD with vaccination overall (all CD) or according to the type of CD sign (optic neuritis or myelitis), for all vaccines overall or separately (influenza, anti-HPV, DTPPHae and anti-hepatitis B vaccines) and whatever the time window considered (24-, 6- and 2-month windows). However, no conclusion could be drawn for anti-hepatitis B vaccine for the 2-month time window and HPV vaccine for the 6-month and 2-month time windows, as fewer than 3 cases or 3 referents were exposed. Results were overall very similar in the subgroup analysis by age group, results reflecting the different vaccines used according to age.

The results of the present study are consistent with the recent findings from several publications [24, 29, 37–41] which showed that none of the following vaccines, namely influenza, HPV, DTPPHae and hepatitis B were associated with an increased risk of CD.

To our knowledge, our study is the largest case-referent study exploring the relation between vaccination and the onset of first CD signs taking into consideration the type of symptom (optic neuritis and/or myelitis) available.

Some findings of our study deserve to further discussion. First, although the association between the influenza vaccine and the risk of CD was not statistically significant, this is the only vaccine yielding OR point estimates above 1 (1.02; 95%CI [0.71–1.47], 1.11; 95%CI [0.64–1.91] and 1.55; 95%CI [0.66–3.64] for 24-, 6-, and 2-month time windows respectively). This was consistent with findings from two other case–control studies that also reported a positive, but not significant, association between the influenza vaccine and the risk of demyelination (i.e. point estimate for multiple sclerosis of 1.6; 95%CI [0.7–3.3] [42] and point estimate for optic neuritis of 1.2; 95%CI [0.60 – 2.3] [27]) whilst also finding point estimates less than 1 for the other types of vaccine. Of note, the adjusted OR for flu vaccine and MS was 0.67 [0.29–1.54] in the persons 45 years old or older, that is the age group with the highest use of this vaccine, systematically recommended and reimbursed from 60 years old and above in the country.

Residual confounding cannot be ruled out to explain findings on the influenza vaccine, as patients are more likely to get vaccinated against the influenza seasonally, when the virus is circulating. As no information on influenza occurrence was collected, the slight CD risk increase observed could be linked either to the seasonal vaccine or to the virus itself circulating at the same time which is known to increase CD risk. Indeed, in another context, researchers have studied together the seasonality, the circulating virus and the influenza vaccination, and showed that the positive association observed is linked to the circulating virus rather than the influenza vaccine itself [43]. Three other studies confirmed this observation, with larger samples [44–46].

Second, regarding anti-HPV vaccine, it seems to have a protective effect against CD (OR = 0.32; 95%CI [0.13–0.81]), as previously observed in three studies [47–49]. A potential explanation of this finding could be the doubts raised by previous case series reports [50–52] amongst physicians preventing prescription of anti-HPV vaccination, when there was a family history of auto-immune disease. This could have led to a’’pre-depletion’’ of susceptible patients, explaining an artificial protective effect of the anti-HPV vaccine against CD. A protective effect of anti-HPV vaccine against CD occurrence cannot however be ruled out, and other investigations could be carried out to further explore this association. The same reasoning can be held for anti-hepatitis B vaccination (potential protective effect given OR = 0.39; 95%CI [0.16–0.94]).

One of the strengths of this study is the very high specificity of CD diagnosis using the PGRx information system. Cases were recruited and reported by expert neurological centres, spread all over the country. A second strength is the attention paid to the accurate identification of the date of CD first signs, which plays a key role in determining the exposure-occurrence relationship and avoiding reverse causality bias. Indeed, the recruiting neurologist and the interviewer had to document the date of first signs through a thorough interview with case patients; they were notably asked for the date of the first signs that led the patient to a consultation with a GP, an ophthalmologist or a neurologist before being referred to the expert centre. A third strength is that the information regarding main exposure and potential risk factors was obtained through a validated and structured standardized telephone interview, administered by trained staff, blinded to the case/referent status. Vaccination status was obtained from patient reports and medical records with high level of agreement between these two sources of information in cases and referents. Indeed, agreement within 3 years before the recruitment date was comprised between substantial for influenza vaccines (prevalence and bias-adjusted kappa [PABAK] = 0.74) to high for 23-valent pneumococcal vaccines (PABAK = 0.98) and HPV vaccines (PABAK = 0.92) [35]. A fourth strength lies in the PGRx information system itself, as it is built to minimize selection bias. Indeed, all participants were first recruited in prospective CD diseases registries and their exposure was assessed without any reference to future studies based on these data such as the current study. Thus, participating physicians and patients were blinded to specific hypotheses regarding vaccination. Fifth, confounding was controlled for key factors both through matching (age, sex, index date and region of residence) and adjustment (family history of auto-immune diseases, region of residence, smoking status and co-vaccinations) so that residual confounding seems unlikely. Finally, in general, referent pools such as that used in this study (PGRx-GP) have been shown to be representative of the general population [53].

Our results should be interpreted in the light of some limitations. First, there was insufficient power to conclude on individual vaccines in the 2-month time window of exposure. Indeed, secondary analysis of each individual vaccine showed a low proportion of CD cases to anti-hepatitis B and anti-HPV vaccines, which have been at the centre of controversies on their alleged risk. The public attitude towards vaccination has a great impact on vaccination rates as illustrated by the drop in anti-hepatitis B vaccine coverage following an initial report of a temporal association between the vaccine and multiple sclerosis onset [54], later followed by a series of negative studies [55, 56]. Second, recall bias cannot be ruled out as information on co-medication, co-vaccination and other potential risk factors was collected through telephone interviewing of participants, going back 24 months earlier. Specifically, differential recall bias (i.e. cases, because of illness, tended to report a more comprehensive exposure than referents) could not be excluded even if it is very unlikely due to efforts to minimize this potential bias using PGRx registries [35].

## Conclusion

No increased risk of CD was observed amongst vaccinated patients. Lower rates of vaccination against HBV and HPV were observed in CD patients compared to referents, which may be due to the reluctance of physicians to vaccinate patients considered at risk of demyelination (family history of auto-immune diseases).

### Supplementary Information

Below is the link to the electronic supplementary material.

Supplementary file1 (DOCX 23 KB)

## Data Availability

Source data may be shared under reasonable request.
