# Supplementary material for: Association between vaccination and the risk of central demyelination: results from a case-referent study
Source: J Neurol. 2023 Jun 23;270(10):4678–86. doi: 10.1007/s00415-023-11822-y (PMC10511379; doi:10.1007/s00415-023-11822-y)
Supplement: Supplementary file 1 — Supplementary file1 (DOCX 23 KB) [file 415_2023_11822_MOESM1_ESM.docx]

| **Table S1. Association between any vaccine, individual vaccine and central demyelination according to the age group [Vaccine within 24 months before ID]** | | | |
| --- | --- | --- | --- |
|  | **Cases n (%)** | **Matched referents n (%)** | **Matched Adjusted**  **OR [95% CI]^*^** |
| **<25 years old** | **n=153** | **n=306** |  |
| Any vaccine | 32 (20.9%) | 105 (34.3%) | 0.46 [0.28 - 0.76] |
| Flu | 6 (3.9%) | 15 (4.9%) | 1.03 [0.38 - 2.84] |
| HPV | 6 (3.9%) | 35 (11.4%) | 0.26 [0.09 - 0.71] |
| DTPPHae | 18 (11.8%) | 52 (17.0%) | 0.70 [0.38 - 1.30] |
| **25-44 years old** | **n=323** | **n=646** |  |
| Any vaccine | 72 (22.3%) | 158 (24.5%) | 0.86 [0.63 - 1.18] |
| Flu | 35 (10.8%) | 65 (10.1%) | 1.17 [0.74 - 1.85] |
| DTPPHae | 37 (11.5%) | 87 (13.5%) | 0.80 [0.53 - 1.19] |
| **≥ 45 years old** | **n=88** | **n=176** |  |
| Any vaccine | 19 (21.6%) | 57 (32.4%) | 0.57 [0.29 - 1.09] |
| Flu | 13 (14.8%) | 37 (21.0%) | 0.67 [0.29 - 1.54] |
| DTPPHae | 6 (6.8%) | 24 (13.6%) | 0.50 [0.19 - 1.31] |

^*^Adjusted OR were computed from conditional logistic regression controlled for family history of autoimmune diseases, region of residence, smoking status and co-vaccinations

Abbreviations: CI, confidence interval; ID, Index date; DTPPHae: Vaccine against diphtheria-tetanus-pertussis-poliomyelitis haemophilus; FLU: Vaccine against Influenza; HBV: Hepatitis B vaccine; HPV: Vaccine against human papillomavirus; Odds Ratios not provided if less than 5 patients in any exposure by disease status cell
